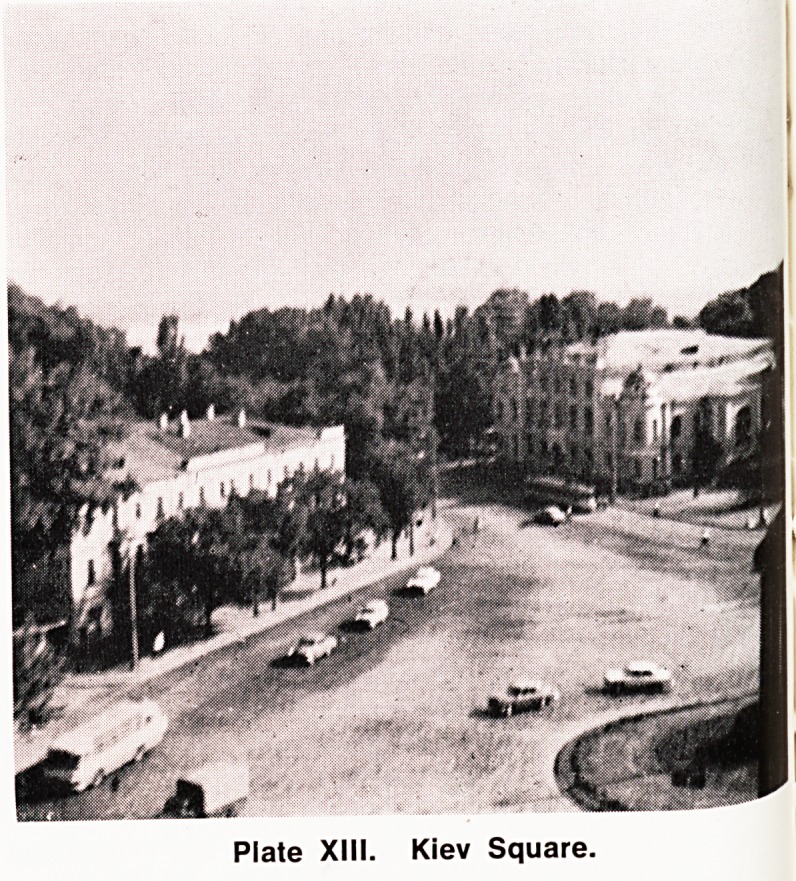# A Visit to the Soviet Red Cross

**Published:** 1970-04

**Authors:** D. W. Wright

**Affiliations:** Ham Green Hospital, Bristol


### A Visit to the Soviet Red Cross

**D. W. Wright, M.B., Ch.B.**
Ham Green Hospital, Bristol

In June 1969 I had the opportunity to visit Russia as a member of a delegation from the British Red Cross Society. The delegation was headed by Lord Inchyra, Chairman of the British Society, and included Mr. Ian Reid, Director of International Affairs, and myself.

The object of the visit was primarily to renew the already excellent relations which exist between the British and Russian Societies, and to compare and contrast their activities, but fortunately I was also given ample opportunity to see some of the country and visit medical services in which I have a special interest.

Our host during the visit was Professor Georgy Miterev, the Chairman of the Soviet Red Cross, who met us, with a reception committee, on the plane when we arrived at Sheremetyevo Airport just outside Moscow. As we walked to the airport lounge, we passed by the Russian equivalent of the Concorde standing gleaming on the tarmac.

One was immediately impressed by the friendly atmosphere into which we were received and, on arriving at the Soviet Red Cross Headquarters in Moscow, we attended a reception at which gifts were exchanged and we were introduced to senior Red Cross officials. During our visit many photographs were taken and, just before we entered the plane to return to England, we were each presented with an album containing photographs commemorating the visit. Though I had learned several phrases in Russian in readiness for the trip, the language was no problem, as many of the Russians spoke good English, and we were helped by excellent lady interpreters. Although Professor Miterev himself spoke no English, one quickly appreciated the gist of what he was saying by his expression and gesticulations and all the time he displayed an enormous amount of energy for a man in his seventies. (Incidentally this equally applied to our own Chairman who was of similar age.)

I quickly became rather envious when I learned of the vast membership of the Russian Red Cross who have over 70 million members out of a population of about 236 million compared with the British membership of nearly 570,000 with our population of about 56 million. However, we soon learned that this large membership was due in no small measure to the fact that there are few other voluntary organisations in Russia for people to join in contrast to the many we have in Britain.

In comparing our activities we discovered that Red Cross Units in Russia are based on one's place of work, e.g. factories, institutions, schools, and the universities. In this country Units are based away from one's work and normally are situated reasonably close to where the members live, and are composed of members with many varied occupations and skills. The Soviet Red Cross has a much closer association with the Official Authorities than we do in this country.

**Plate XI. The Kremlin. Cathedral of the Assumption.**

Our visit, starting at Moscow, gave us an opportunity to visit the Kremlin (built 1147 with the walls added in 1339) where we attended a reception, and to see some of the objects of historical interest within its walls including the Cathedral of the Assumption (built 1475-1479) (Plate XI), the Tsar's Cannon (built in the 16th Century; weighing 40 tons and 16 feet in length), and the Tsar's Bell (cast 1733-1735, and weighing 200 tons) (Plate XII). In the evening we were able to watch the changing of the guard outside Lenin's Tomb. One evening we went to a concert in a modern hall built within the Kremlin and were surprised that one of the items included a young pop-group who sang "Irene" in English (not, I might add for our special benefit!).

One thing which did surprise me in Moscow was that the Red Square, though very impressive did not

**Plate XII. Inside the Kremlin. The Tsar's Bell.**

seem quite as large as it appears to be when watching the parades of the Soviet Armed Forces on British television, and as our hotel in Moscow overlooked the Kremlin, we sometimes took a walk in the Square before or after mealtimes.

While in Moscow I had an opportunity to visit the Institute of Cardiology where I spent some time in their Coronary Care Unit which, though housed in an old building, allowed plenty of space for each patient, much more than some of the units I have seen in this country. The Professor in charge spoke perfect English and his chief assistant had spent some time working and visiting cardiology units in Great Britain. The Unit was divided into four sections, the patients' movements within the sections depending on the severity of their condition. Though there was not a patient using it while I was there, I saw an interesting piece of apparatus which is being used to try to assist the coronary circulation in cardiogenic shock and was shown some tracings demonstrating an apparent increase in coronary blood flow taken while this instrument was being used.

On the third day of the visit I woke up with a cold, and on asking for some aspirin was taken to the nearest Polyclinic, where I not only received treatment in various departments, but also had an opportunity to see the working of such a unit, which to me seemed to be a sort of half-way house between a British health centre and a hospital, as different specialists (not quite the equivalent of consultants in this country) worked there and many investigations and treatments could be carried out, including radiology, physiotherapy, pathology, and minor surgery. The Russians have a large paramedical service with medical aides called "Felschers" (who have a four year training) performing many of the duties which only doctors would, at present, do in this country. I am happy to say that my cold was quickly cured, due actually in no small measure to treatment rendered by my colleague, Mr. Ian Reid, who being used to travelling all over the world in his capacity as Director of International Affairs, always carried a kit of various "potions"—a case of a potential patient treating the doctor!

We next travelled by over-night train to the next largest city, Leningrad. During the long journey, in a very comfortable sleeper, we were frequently supplied with hot, sweet lemon tea, without milk, which was "brewed" up on a coke fire in the corner of each carriage by an attendant.

In Leningrad, after being greeted by the local Red Cross Chairman (incidentally all such chairmen that we met were women of considerable capabilities) we were able to see much of the city and its fine treasures, including the museum called the Hermitage. We were amazed at the way in which the city has been rebuilt to the original style that existed before it was almost completely destroyed by the Germans during the war. We then travelled by hydrofoil up river towards the Gulf of Finland and visited the Tsar's Winter Palace, parts of which were still under reconstruction following the damage sustained in the war. The Russian people are constant visitors to the museum.

**Plate XIII. Kiev Square.**

From Leningrad we flew to Kiev (Plate XIII) the third largest city, and then travelled by road into the Ukraine where we spent a day on a collective farm, appropriately called Friendship. It was from this farm that there came one of the great Resistance Leaders who led the people during the time the Ukraine was overrun by the Germans. The farm covered about 14,000 acres and contained a very well run nursery to which allowed both husband and wife to be free to work on the farm during the day. Medical services on the farm were provided by the Felschers, with supervision by a District Doctor, who was responsible for the whole area. During the day's visit when we saw some of the work of the farm and visited the workers' homes, we were invited into one of their houses for lunch, which we shared with the family, and tasted many of their traditional dishes. We then returned by plane to Moscow. Travel within Russia seemed to be fairly cheap, presumably being subsidised by the Government, the cost of the various forms of travel being fairly similar, whether by train, bus, metro, taxi, or air, this being, perhaps, one of the reasons why only a few people at present own their own cars.

While in Moscow we had an opportunity of visiting a Liquor Plant, primarily to watch the works first-aid team in action with a simulated incident, but also to sample the many different varieties of vodka produced by the plant and exported all over the world. Part of our time was taken up in visiting an Old People's Home, on the outskirts of the city, occupied by retired actors and actresses who were encouraged to look after themselves as much as possible; in fact, their equivalent of our House Committee was composed of the residents with the Resident Doctor, the Director as Adviser. The married couples lived in flatlets.

A most interesting time was spent on a visit to the Moscow State University, an enormous building housing over 16,000 students and staff with its own Polyclinic and Sanatorium. The Red Cross was very active within the University. In this country only some of the Universities have Red Cross groups. Bristol has a very active one.

Before leaving Moscow we were invited to a luncheon party given by the British Ambassador, Sir Duncan Wilson and his wife in the Embassy, an English style house which looks out across the river facing the Kremlin, and by an amazing coincidence I sat next to a lady who has a relative whom I know living in Bristol, and who hopes to send her daughter to a school here. It is certainly a small world!

From our visit we found the work of the Soviet Red Cross very similar to that of our own Society, namely, first aid, home nursing, and welfare, though some of the work done by its members would probably be done by paid personnel in this country. There was great stress on personal hygiene, starting in the nurseries, and on preventive medicine, the Russians believing in regular examination and screening of a large proportion of the population. Professor Miterev, our host, was particularly interested in this aspect of medicine, having been the People's Commisar for Health in Moscow during the last war; he has been commissioned to write the Official story of the Care of the Health of the Citizens of Moscow during that period with particular reference to epidemic control.

As to the country, I was impressed by its cleanliness (there was little or no litter lying about on the pavements or roads—due, no doubt, in no small measure, to the laws of the land), its wide roads with good lane discipline, and the friendliness of the people we met. I came away with the feeing that the visit had been of considerable mutual benefit and that the Soviet Red Cross's desire to help other people was similar to that of our own Society and of others throughout the World in an Organisation which has a total membership of 220 million with 112 National Societies.

**Acknowledgement**

I would like to express my grateful thanks to the Bristol Red Cross Society for inviting me to join the delegation and to the Soviet Red Cross for their wonderful hospitality.

## Figures and Tables

**Plate XI. f1:**
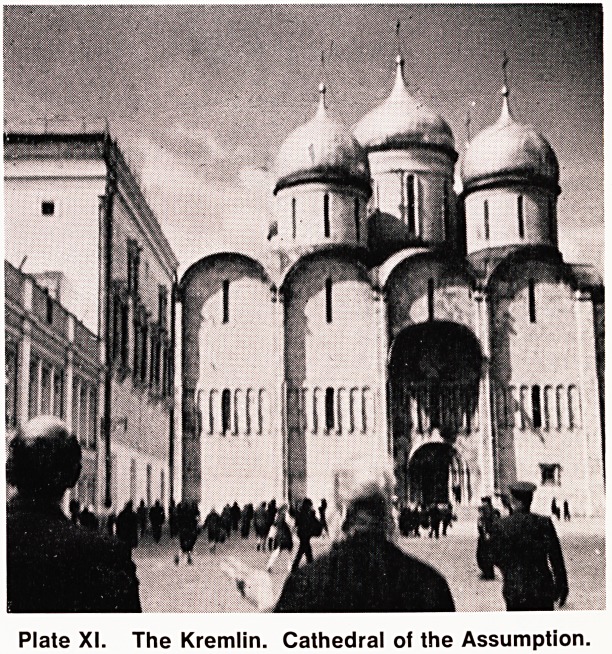


**Plate XII. f2:**
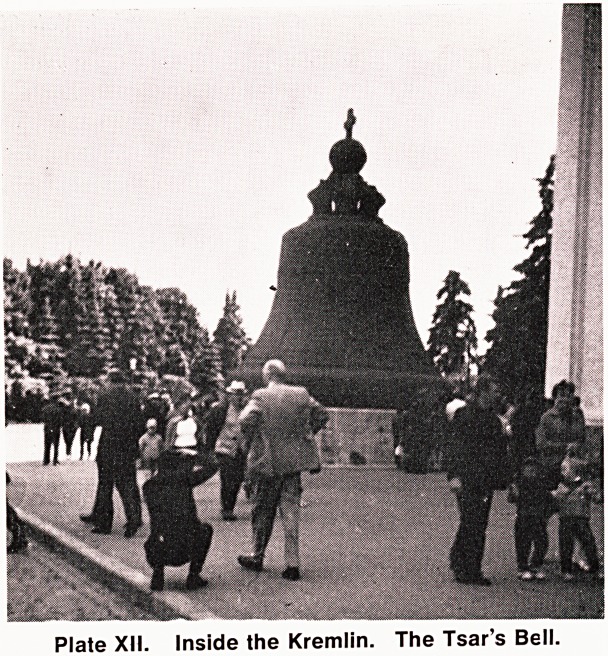


**Plate XIII. f3:**